# High follicle‐stimulating hormone levels accelerate cartilage damage of knee osteoarthritis in postmenopausal women through the PI3K/AKT/NF‐κB pathway

**DOI:** 10.1002/2211-5463.12975

**Published:** 2020-09-21

**Authors:** Yaping Liu, Mengqi Zhang, Dehuan Kong, Yan Wang, Jian Li, Wenjuan Liu, Yilin Fu, Jin Xu

**Affiliations:** ^1^ Department of Endocrinology Shandong Provincial Hospital Cheeloo College of Medicine Shandong University Jinan China; ^2^ Shandong Provincial Key Laboratory of Endocrinology and Lipid Metabolism Jinan China; ^3^ Institute of Endocrinology and Metabolism Shandong Academy of Clinical Medicine Jinan China; ^4^ Department of Endocrinology Jining No. 1 People’s Hospital Jining China; ^5^ Department of Endocrinology Taian City Central Hospital Taian China; ^6^ Department of Bone and Joint Surgery Jining No. 1 People’s Hospital Jining China; ^7^ Department of Magnetic Resonance Imaging Jining No. 1 People’s Hospital Jining China; ^8^ Department of Endocrinology Shandong Provincial Hospital Affiliated to Shandong First Medical University Jinan China

**Keywords:** aggrecan, follicle‐stimulating hormone, knee osteoarthritis, PI3K/AKT/NF‐κB pathway, postmenopausal women, type II collagen

## Abstract

Osteoarthritis is the main cause of pain and disability in the elderly, with the most commonly affected joint being the knee. The prevalence of knee osteoarthritis (KOA) is significantly increased in postmenopausal women, although the mechanisms underlying KOA remain unclear. The present study aimed to investigate the association between follicle‐stimulating hormone (FSH) and postmenopausal women with KOA aged between 50 and > 70 years, as well as explore its underlying molecular mechanisms. Here, we report that the 50–60 years age group had the highest level of serum FSH. Compared to the low FSH group (< 40 mIU·mL^−1^) in the same age group, the high FSH group (> 40 mIU·mL^−1^) showed more severe cartilage damage. Furthermore, phosphorylated (p)‐phosphoinositide 3‐kinase (PI3K)/PI3K, p‐AKT/AKT and p‐nuclear factor kappa B (NF‐κB)/NF‐κB levels were significantly higher in the high FSH group compared to the low FSH group. Immunofluorescence experiments showed that FSH stimulation promoted the translocation of NF‐κB p65 into the nucleus, and decreased type II collagen and aggrecan in ATDC5 cells. Moreover, we used western blotting in ATDC5 cells to demonstrate that FSH decreased type II collagen and increased p‐PI3K/PI3K, p‐AKT/AKT, p‐NF‐κB/NF‐κB and p‐IKB/IKB in a concentration‐dependent manner. Our results suggest that increased FSH levels are associated with KOA for postmenopausal women aged 50–60 years and that high FSH levels might damage the cartilage tissues through the PI3K/AKT/NF‐κB pathway.

## Graphical abstract

Knee osteoarthritis (KOA) is prevalent in postmenopausal women and a cause of pain and disability in elderly populations. Here, we report high follicle‐stimulating hormone (FSH) levels across postmenopausal female KOA patients aged 50–60 years. We speculate FSH might damage cartilage tissues through the phosphoinositide 3‐kinase/AKT/nuclear factor kappa B pathway. Our findings suggest that FSH modulation holds promise as a novel treatment for postmenopausal female KOA patients aged 50–60 years.

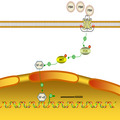

**Abbreviations**
ANOVA: one‐way analysis of variance
BMI: body mass index
E2: estradiol
ECM: extracellular matrix
FSH: follicle‐stimulating hormone
HDL‐C: high‐density lipoprotein cholesterol
KOA: knee osteoarthritis
LDL‐C: low‐density lipoprotein cholesterol
LH: luteinizing hormone
PBS: NaCl/Pi
OA: osteoarthritis
TC: total cholesterol
TG: triglycerides

As a chronic degenerative joint disease, osteoarthritis (OA) is the main cause of pain and disability in the elderly, which seriously affects the life quality of patient' [1, 2, 3]. The knee is the most common affected joint in OA [4]. At present, controlling symptoms is the main treatment for knee osteoarthritis (KOA). However, analgesics such as non‐steroidal anti‐inflammatory drugs can cause side effects, including gastrointestinal disorders. If the disease progresses, knee replacement surgery is needed but, for older patients, the surgery risk is high. The onset of KOA has been found to be affected by many factors, such as aging [5, 6, 7]. Especially, the prevalence of KOA is significantly increased in postmenopausal women [8]. However, the underlying mechanisms remain to be clarified.

Follicle‐stimulating hormone (FSH), a pituitary glycoprotein, plays an important role in the maturation and development of follicles. In addition to decreased estrogen, high FSH levels have been found after menopause [9]. FSH has been shown to accelerate osteoclast formation through the Gi2α‐coupled FSH receptor (FSHR) [10]. Furthermore, FSHR stimulation activates the AKT/nuclear factor kappa B (NF‐κB) pathway to produce osteoclasts [11]. Increasing research suggests that FSH is associated with osteoporosis in menopausal women [12]. A previous study has found that FSHR protein is located on the human chondrocyte membrane [13]. Postmenopausal osteoporosis and OA are both age‐related diseases; however, no reports are available concerning the increase in FSH affecting the occurrence and progress of KOA. Thus, we analyzed the correlation between FSH and postmenopausal KOA women in this study.

Phosphoinositide 3‐kinase (PI3K)/AKT/NF‐κB has been found to be involved in the degeneration and destruction of OA articular cartilage. For example, inhibition of PI3K/AKT/mammalian target of rapamycin pathway could induce articular chondrocyte autophagy and suppress inflammatory processes in OA rats [14]. Allicin alleviates the progression of OA by inactivating the PI3K/AKT/NF‐κB pathway [15]. Blocking PI3K/AKT signaling can reduce post‐traumatic OA or tumor necrosis factor‐α‐induced OA [16, 17]. Thus, blocking this pathway could be a promising treatment strategy for KOA.

In the present study, we investigated whether a high FSH level was significantly associated with postmenopausal women with KOA aged 50–60 years. Moreover, FSH might promote articular cartilage destruction through the PI3K/AKT/NF‐κB pathway.

## Materials and methods

### Patients and specimen collection

In the present study, we recruited 215 postmenopausal women aged over 50 years who underwent total knee arthroplasty, unicondylar knee arthroplasty or conservative treatment in the bone and joint surgery ward of Jining No. 1 People's Hospital from January 2016 to October 2018. All patients were divided into three groups: 50–60 years age group (*n* = 79), 61–70 years age group (*n* = 88) and > 70 years age group (*n* = 48). In the 50–60 years age group, all patients were classified into a high FSH group (FSH> 40 mIU·mL^−1^; *n* = 45) and a low FSH group (FSH < 40 mIU·mL^−1^; *n* = 34). More importantly, all subjects had low estrogen levels. Clinical characteristics, including menopausal age, history of KOA, height, weight, body mass index (BMI) and physical examination, were recorded. The inclusion criteria of the study were based on the OA diagnosis and treatment guidelines developed by the Chinese Orthopaedic Association Joint Surgery Group in 2018, a Western Ontario and McMaster Universities Osteoarthritis Index (WOMAC) score of 12 points, X‐ray films and knee magnetic resonance T2‐positioning imaging. The WOMAC scale was used to assess the clinical symptoms of KOA, including three dimensions and 24 items, namely pain (five items), stiffness (two items), and physical function (17 items). All items were rated on a scale of 0 (asymptomatic/ability) to 10 (maximum symptoms/ability disorder). A score of < 80 was considered mild, 80–120 was moderate and > 120 was severe. The higher the score, the more severe the symptoms of KOA. A TrioTim 3.0T magnetic resonance scanner (Siemens, Munich, Germany) was used for T2 mapping imaging. The same individual drew two regions of interest in the corresponding regions of the T2 map and averaged them. Exclusion criteria were: (a) patients with congenital malformation of knee or OA as a result of recent trauma; (b) patients with signs of infection; (c) patients who had received a history of steroid use within the last 3 months; and (d) severe impaired liver or renal function, hyperthyroidism, hyperparathyroidism, diabetes, dyslipidemia, or rheumatoid disease that may affect bone metabolism.

Human cartilage tissues were obtained from postmenopausal female patients with KOA aged 50–60 years in the bone and joint surgery ward of Jining No. 1 People's Hospital. All patients provided their written informed consent. The study met the principles of the Declaration of Helsinki and was approved by the Ethics Committee of Jining No. 1 People’s Hospital (JNDYRM‐2016‐017).

### Detection of human serological indices

Fasting serum specimens were collected. Serum FSH, luteinizing hormone (LH) and estradiol (E2) were detected by electrochemical luminescence with a Cobas e 601 immunoassay analyzer (Roche, Basel, Switzerland). Fasting blood glucose, triglycerides (TG), total cholesterol (TC), high‐density lipoprotein cholesterol (HDL‐C) and low‐density lipoprotein cholesterol (LDL‐C) were detected by enzyme colorimetry using a Cobas 8000 automatic biochemical analyzer (Roche).

### Safranin O‐fast green staining

Bone blocks in the femoral condyle loading area of postmenopausal female patients with KOA aged 50–60 years were collected for safranin O‐fast green staining. The osteochondral tissues were cut perpendicular to the articular surface with a bone knife approximately 1.0 × 1.0 × 1.0 cm (length × width × depth) and then soaked in 10% formalin solution for 24 h. EDTA decalcification solution (slow decalcification) lasted for approximately 1 month, during which the decalcification solution of EDTA was changed every 2 days. When the bones could easily be penetrated with the syringe needle perpendicular to the joint surface, this indicated that the decalcification was completed. After conventional paraffin embedding, longitudinal section (thickness 5 µm), saffron/fast green staining was performed, followed by microscopic examination.

### Cell culture

The murine chondrogenic ATDC5 cell lines were purchased from the Chinese Academy of Science (Shanghai, China), which were cultured in Eagle's minimum essential medium (Gibco, Gaithersburg, MD, USA) plus 10% fetal bovine serum, 2 mm·L^−1^ glutamine and 100 U·mL^−1^ penicillin‐streptomycin in a humidified incubator with 5% CO_2_ at 37 °C. The culture medium was changed every 2 days. The morphology and growth density of ATDC5 cells were observed under an inverted phase‐contrast microscope. The cells were laid in a single layer on the bottom of the Petri dish, and there was adherent aggregation, which was triangular or polygonal. When the density of cells reached 80–90% in the culture dish, complete culture mediums were sucked out and discarded. The basic culture medium without serum was added to starve the cells for 2 h. Subsequently, the cells were incubated with FSH (recombinant mouse FSH α/β; R&D Systems, subsidiary of Bio‐Techne, Minneapolis, MN, USA; 8576‐fs‐010) at concentrations of 0, 9, 30 and 90 ng·μL^−1^ for 30 min.

### Protein extraction

The frozen human cartilage tissues that were stored in a refrigerator at −80 °C were removed and placed on the ice. The tissues were weighed to 80 mg and ground in liquid nitrogen. The powder was collected and added to a new Eppendorf tube, followed by 600 µL of RIPA lysis buffer (RIPA + phenylmethanesulfonyl fluoride + phosphatase inhibitor A + B ratio was 98 : 1 : 0.5 : 0.5). It was left on ice for 20 min and then sonicated. Then, the samples were centrifuged at 12 000 ***g***. for 15 min at 4 °C. Three layers appeared after centrifugation and the middle transparent liquid was absorbed into a new Eppendorf tube. A one‐third protein volume of loading buffer was added to the protein sample, and the protein was denatured in a water bath at 99.9 °C for 10 min. Tissue protein concentration was determined by the bicinchoninic acid method.

After the stimulation of cells with different concentrations of FSH for 30 min, the cell culture dishes of each group were placed on the ice, the basic culture medium was sucked off, and the cells were washed with ice NaCl/P_i_ (PBS) three times, and then the PBS was sucked clean. The RIPA lysate was put into each group of cell culture dishes at the same time, and the configuration method was as described above. Cells were scraped one dish after another, collected at the bottom of the dish and transferred into a new Eppendorf tube. The following steps were consistent with the human cartilage protein extraction as described previously.

### Western blot analysis

The proteins were separated by SDS/PAGE with 10% polyacrylamide gels and transferred to polyvinylidene fluoride membranes (Millipore, Billerica, MA, USA). The membranes were incubated with primary antibodies at 4 °C overnight after being blocked with 0.5% non‐fat milk powder for 1 h. Primary antibodies were as: anti‐type II collagen (ab34712; dilution 1 : 5000; Abcam, Cambridge, UK), anti‐phospho‐PI3K (ab182651; dilution 1 : 1000; Abcam), anti‐phospho‐NF‐κB (ab76302; dilution 1 : 10 000; Abcam), anti‐NF‐κB (ab16502; dilution 1 : 2000; Abcam), anti‐PI3K (cst5405; dilution 1 : 1000; Cell Signaling Technology, Boston, MA, USA), anti‐phospho‐AKT (cst4060S; dilution 1 : 2000; Cell Signaling Technology), anti‐AKT (cst4691S; dilution 1 : 1000; Cell Signaling Technology), anti‐phospho‐IKBα (cst9246; dilution 1 : 1000; Cell Signaling Technology), anti‐IKBα (cst4812; dilution 1 : 1000; Cell Signaling Technology) and anti‐GAPDH (abs83030; dilution 1 : 5000; Absin Bioscience Inc, Shanghai, China). After incubation with anti‐rabbit or anti‐mouse secondary antibodies at room temperature, the immune complexes were detected on a shaker. Membrane imaging was performed using an Alpha FluroChem Q imaging analysis system (Alpha Innotech, San Leandro, CA, USA). GAPDH was used as a control.

### Immunofluorescence

ATDC5 cells were seeded on a medium containing fetal bovine serum for 1–2 days. The complete medium was then aspirated and discarded. The cells were starved for 2 h by adding serum‐free alkaline medium. Recombinant mouse FSHα/β was used to incubate cells at a concentration of 0 and 90 ng·µL^−1^ for 24 h. After washing with cold PBS solution 1–2 times, the cells were fixed on ice with 4% paraformaldehyde for 15 min. After washing again, the cells were infiltrated with 0.5% Triton‐100 for 20 min, and then blocked with 10% goat serum for 30 min. Next, the cells were incubated with primary antibodies at 4 °C overnight, including anti‐NF‐κB (ab16502; dilution 1 : 1000), anti‐type II collagen (ab34712; dilution 1 : 200) and anti‐Aggrecan (ab3778; dilution 1 : 50). Then, the cells were incubated with fluorescein isothiocyanate‐conjugated goat anti‐rabbit or goat anti‐mouse IgG (Alexa Fluor; Life Technologies, Grand Island, NY, USA) at 37 °C. Nuclei were stained with 4',6‐dimidyl‐2‐phenylindole (Vector Laboratories, Inc. Burlingame, CA, USA). The immunofluorescence results were observed under a fluorescence microscope (LSM780; Carl Zeiss, Oberkochen, Germany).

### Statistical analysis

Statistical analyses were carried out using prism, version 8.0 (GraphPad Software Inc., San Diego, CA, USA). All experiments were repeated at least three times. Data were expressed as the mean ± SD. Student's *t*‐test was used to compare the differences between two groups, whereas one‐way analysis of variance (ANOVA) was used for multiple comparisons. *P* < 0.05 was considered statistically significant.

## Results

### FSH level is significantly decreased as postmenopausal female KOA patients age

**Fig. 1:**
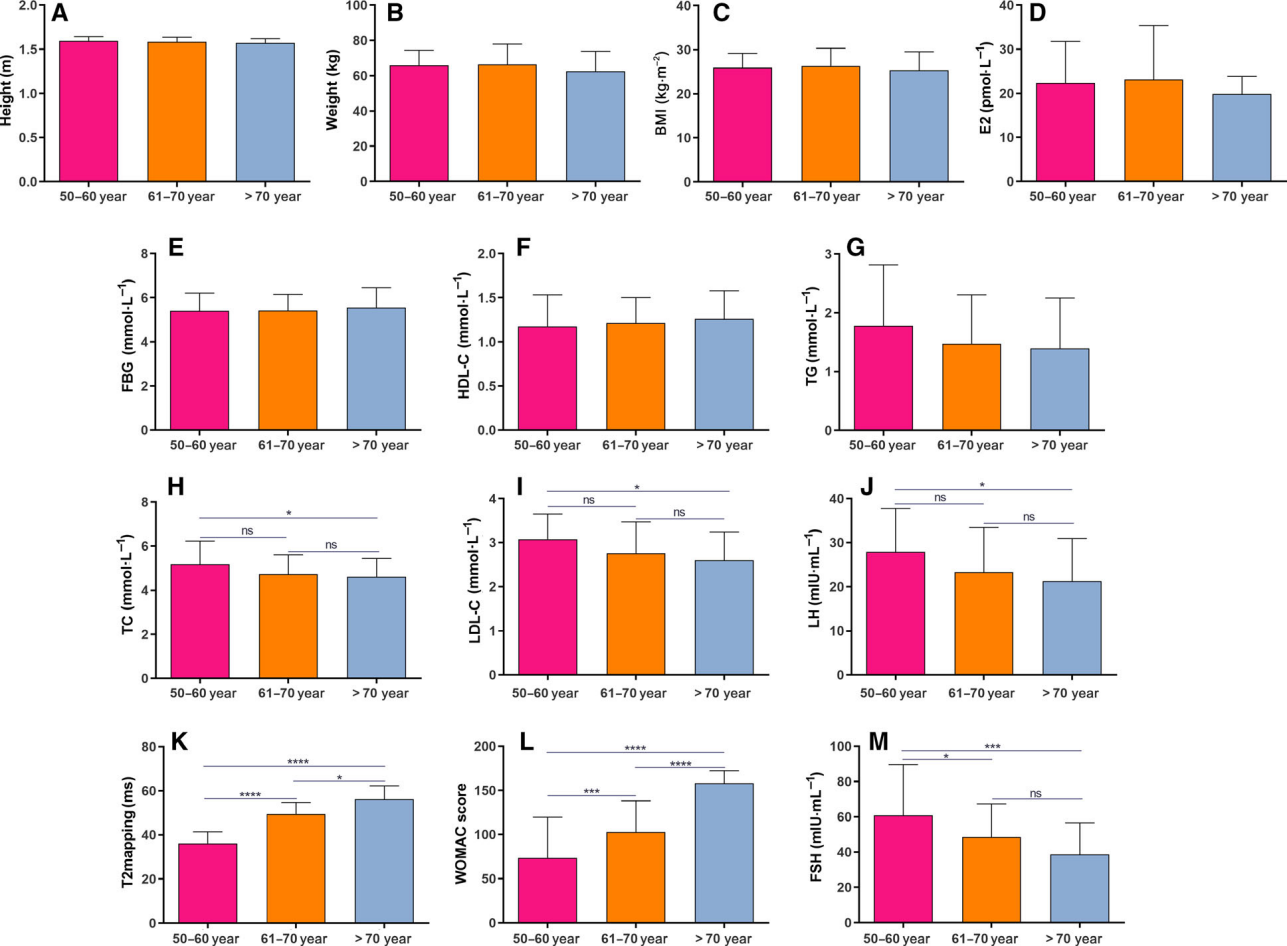
FSH level is significantly decreased as postmenopausal female KOA patients age. The levels of height (A), weight (B), BMI (C), E2 (D), fasting blood glucose (E), HDL‐C (F), TG (G), TC (H), LDL‐C (I), LH (J), average T2 (K), WOMAC scores (L) and FSH (M) in the 50–60 years age group (*n* = 79), 61–70 years age group (*n* = 88) and > 70 years age group (*n* = 48). Data are expressed as the SEM. Multiple comparisons are presented using one‐way ANOVA. *P < 0.05; ***P < 0.001 and ****P < 0.0001. ns, no statistical significance.

In the present study, postmenopausal female patients with KOA were divided into three groups: 50–60 years age group (*n* = 79), 61–70 years age group (*n* = 88) and > 70 years age group (*n* = 48). The clinical characteristics are listed in Table 1. The results showed that there was no significant difference in height, weight, BMI, E2, HDL‐C and TG among the three groups (Fig. 1A‐G). The levels of TC, LDL‐C and LH in patients 50–60 years were significantly increased than those in patients > 70 years (Fig. 1H‐J). As the age increased, WOMAC scores and average T2 values significantly increased in the three groups (Fig. 1K,L). More importantly, we found that FSH levels were significantly decreased with increasing age (Fig. 1M). These results indicated that KOA was an aging disease.

**Table 1:** Clinical characteristics of postmenopausal female patients with KOA.

Clinical characteristics	50–60 years age group (*n* = 79)	61–70 years age group (*n* = 88)	> 70 years age group (*n* = 48)
BMI (kg·m^−2^)	25.91 ± 3.27	26.24 ± 4.12	25.33 ± 4.23
E2 (pm)	22.33 ± 9.44	23.16 ± 12.18	19.84 ± 4.00
FSH (mIU·mL^−1^)	60.91 ± 28.78	48.46 ± 18.93	38.65 ± 18.01
HDL‐C (mm)	1.17 ± 0.36	1.22 ± 0.29	1.26 ± 0.32
LDL‐C (mm)	3.08 ± 0.57	2.76 ± 0.72	2.60 ± 0.65
LH (mIU·mL^−1^)	27.95 ± 9.80	23.35 ± 10.10	21.25 ± 9.70
T2 mapping (ms)	36.19 ± 5.21	49.43 ± 5.36	56.16 ± 6.15
TC (mm)	5.17 ± 1.05	4.73 ± 0.88	4.62 ± 0.83
TG (mm)	1.78 ± 1.04	1.47 ± 0.83	1.40 ± 0.86
WOMAC score	73.49 ± 46.20	102.80 ± 35.49	158.1 ± 14.34
Height (m)	1.60 ± 0.05	1.57 ± 0.05	1.57 ± 0.05
Weight (kg)	65.87 ± 8.62	66.27 ± 11.72	62.57 ± 11.33
Fasting blood glucose (mm)	5.41 ± 0.80	5.42 ± 0.73	5.56 ± 0.90

**Fig. 2:**
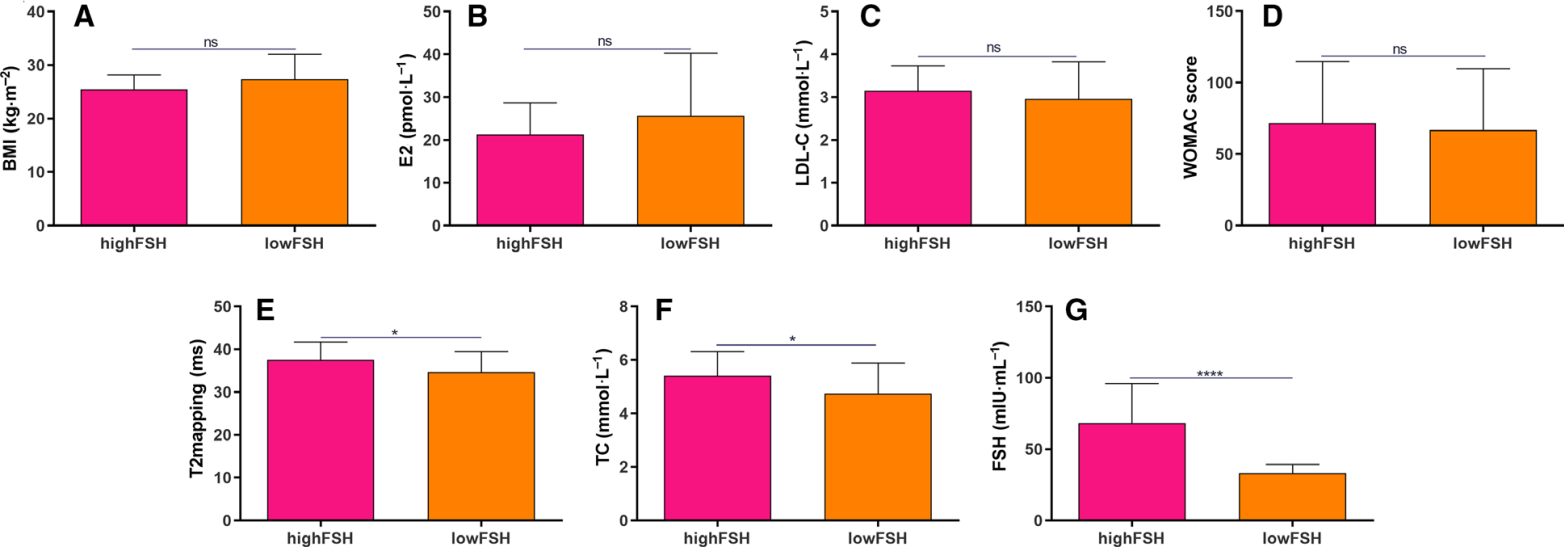
The difference between high FSH and low FSH group. The levels of BMI (A), E2 (B), LDL‐C (C), WOMAC scores (D), average T2 (E), TC (F) and FSH (G) in the high FSH group (*n* = 45) and low FSH group (*n* = 34). Data are expressed as the SEM. Student's *t*‐test was used to compare differences between two groups. *P < 0.05 and ****P < 0.0001. ns, no statistical significance.

In the 50–60 years age group, all patients were classified into a high FSH group (FSH > 40 mIU·mL^−1^; *n* = 45) and a low FSH group (FSH < 40 mIU·mL^−1^; *n* = 34). We found that there was no significant difference in BMI, E2, LDL‐C and WOMAC scores between the two groups (Fig. 2A‐D). Furthermore, average T2 and TC levels were both significantly higher in the high FSH group compared to the low FSH group (Fig. 2E,F). As expected, FSH had a higher level in the high FSH group compared to the low FSH group (Fig. 2G).

### Pathological changes of articular cartilage tissues in postmenopausal female KOA patients with low and high FSH

**Fig. 3:**
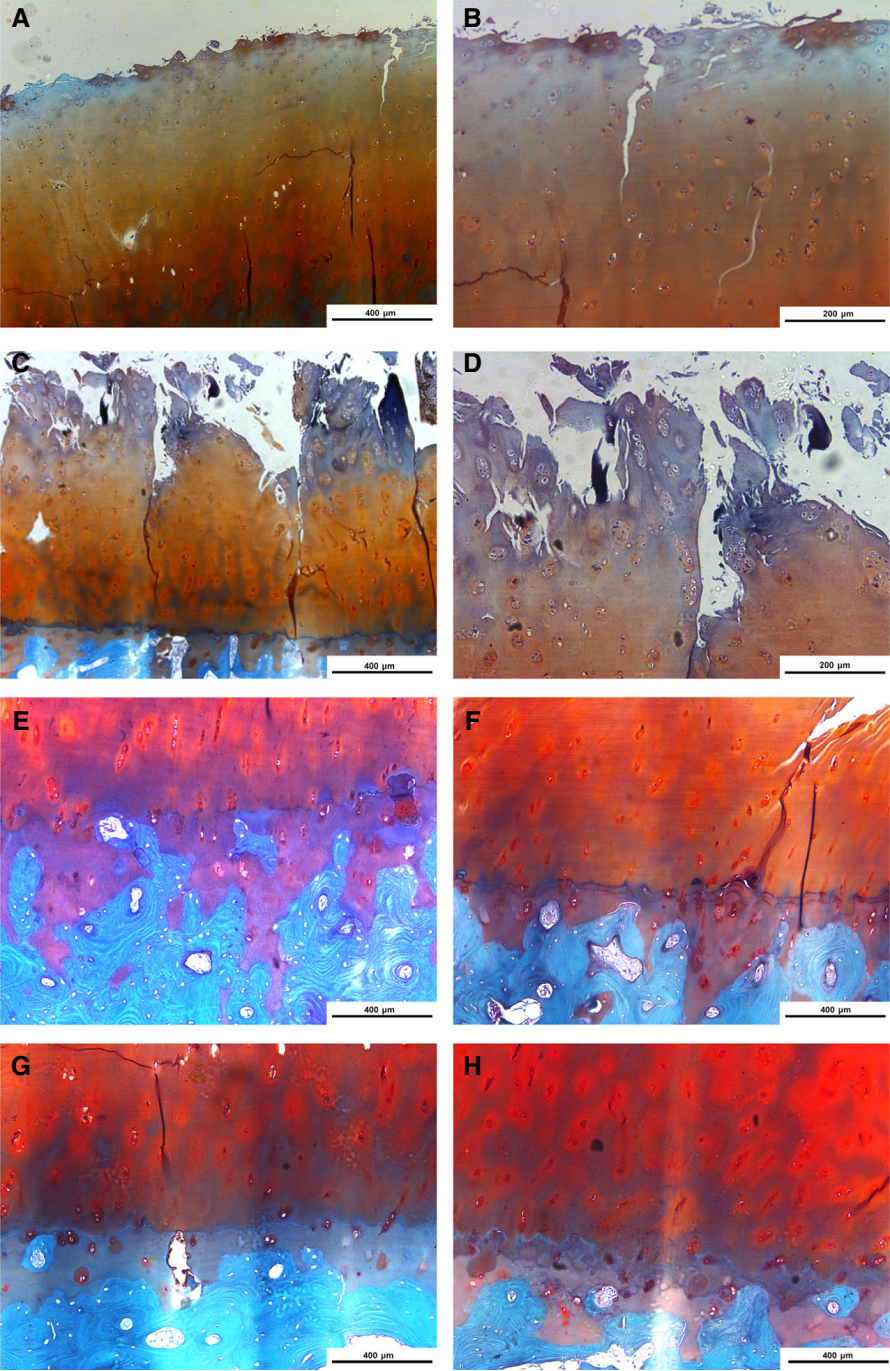
Pathological changes of articular cartilage tissues in postmenopausal female KOA patients with low FSH and high FSH according to saffran/fast green. (A, B) Low FSH (A: ×50, B: ×100). (C, D) High FSH group (C: ×50, D: ×100). The images in (B) and (D) are magnified versions of the images in (A) and (C), respectively. (E‐H) High FSH (×50). Scale bar: 400 μm in (A), (C), (E), (F), (G) and (H) or 200 μm (B) and (D).

We further observed the pathological changes of cartilages in postmenopausal female KOA patients with low and high FSH by saffran/fast green staining. For low FSH, superficial cracks could be found on the surface of cartilage, with a slightly disordered arrangement of chondrocytes, clustered in the transitional layer (Fig. 3A,B). We also found that dehydrated, pyretic and necrotic chondrocytes were at the junction of the transitional layer and the radiation layer, forming defect areas, and multiple voids and sockets appeared on the surface, with relatively complete tidal lines and a fair distribution density of cells (Fig. 3A,B). Furthermore, the numbers of cells in the low FSH group were increased compared to those in the high FSH group. Regarding the pathological changes of cartilages in the high FSH, the surface of cartilage was rough and multiple cracks appeared, extending from the surface of cartilage to the radiation layer (Fig. 3C,D). Moreover, loose network connections could be seen in the cracks, with the phenomenon of chondrocyte clustering. The number of chondrocytes was significantly decreased, and the four‐layer structure was disordered, which was not easy to distinguish (Fig. 3C,D). We further observed the pathological changes of cartilages of postmenopausal female KOA patients with high FSH under a 50× microscope. First, tidal markers were duplicated, drifted and the gap expanded. Multiple tidal markers and significant thickening of the calculation layer were found. Second, the calculation layer thickened as the blood vessels. Third, breaks and lack of tide marks were observed. Fourth, the calcified layer and deep cartilage were defective and the subchondral bone was directly exposed (Fig. 3E‐H).

### FSH is significantly associated with activation of PI3K/AKT/NF‐κB pathway for postmenopausal female KOA patients

**Fig. 4:**
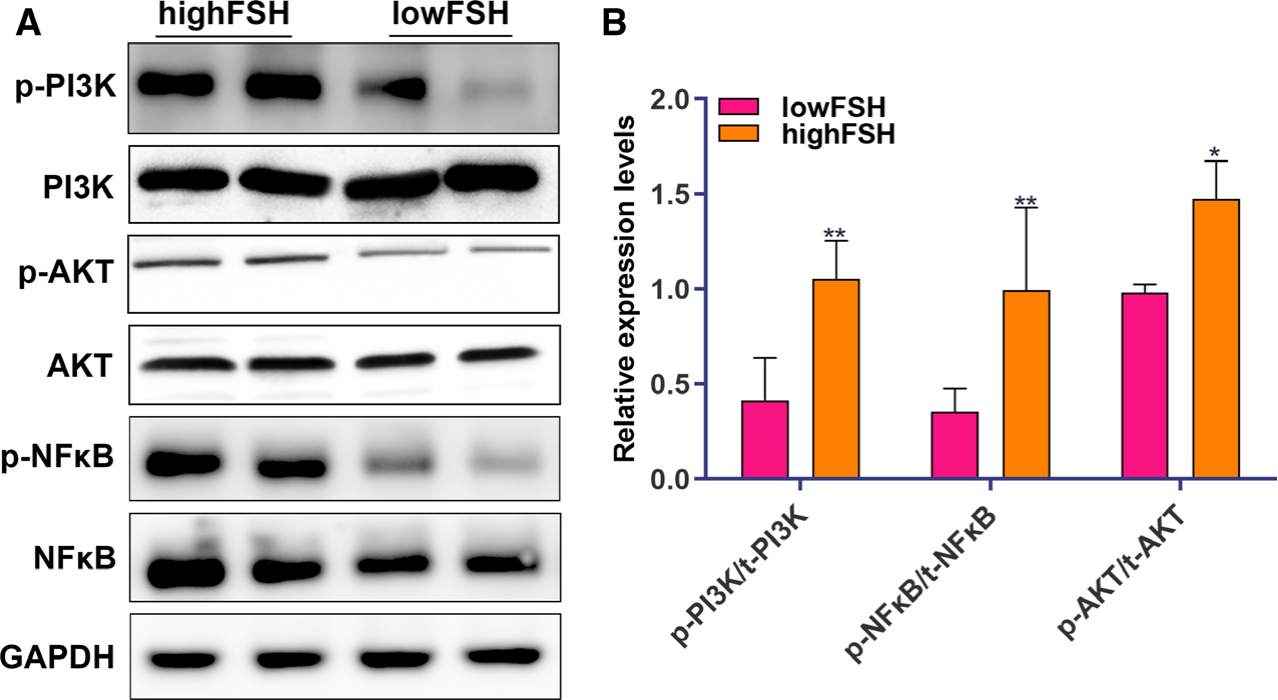
FSH is significantly associated with activation of PI3K/AKT/NF‐κB pathway for postmenopausal female KOA patients. (A) Representative images of western blotting. (B) The expression levels of p‐PI3K/PI3K, p‐AKT/AKT and p‐NF‐κB/NF‐κB. The relative expression given in the *y*‐axis was normalized to GAPDH (*n* = 79.) Data are expressed as the SEM. Student's *t*‐test was used to compare differences between two groups. *P < 0.05 and **P < 0.01.

The expression levels of phosphorylated (p)‐PI3K, PI3K, p‐AKT, AKT, p‐NF‐κB and NF‐κB were detected in articular cartilage tissues of postmenopausal female KOA patients with low FSH (< 40 mIU·mL^−1^) and high FSH (> 40 mIU·mL^−1^) using western blotting. Compared with the low FSH group, the expression levels of p‐PI3K/PI3K, p‐AKT/AKT and p‐NF‐κB/NF‐κB were significantly higher in the high FSH group (Fig. 4A,B). These results indicated that high FSH was significantly associated with activation of PI3K/AKT/NF‐κB pathway for postmenopausal female KOA patients.

### FSH could decrease type II collagen and aggrecan expression and activate PI3K/AKT/NF‐κB pathway in chondrocytes

**Fig. 5:**
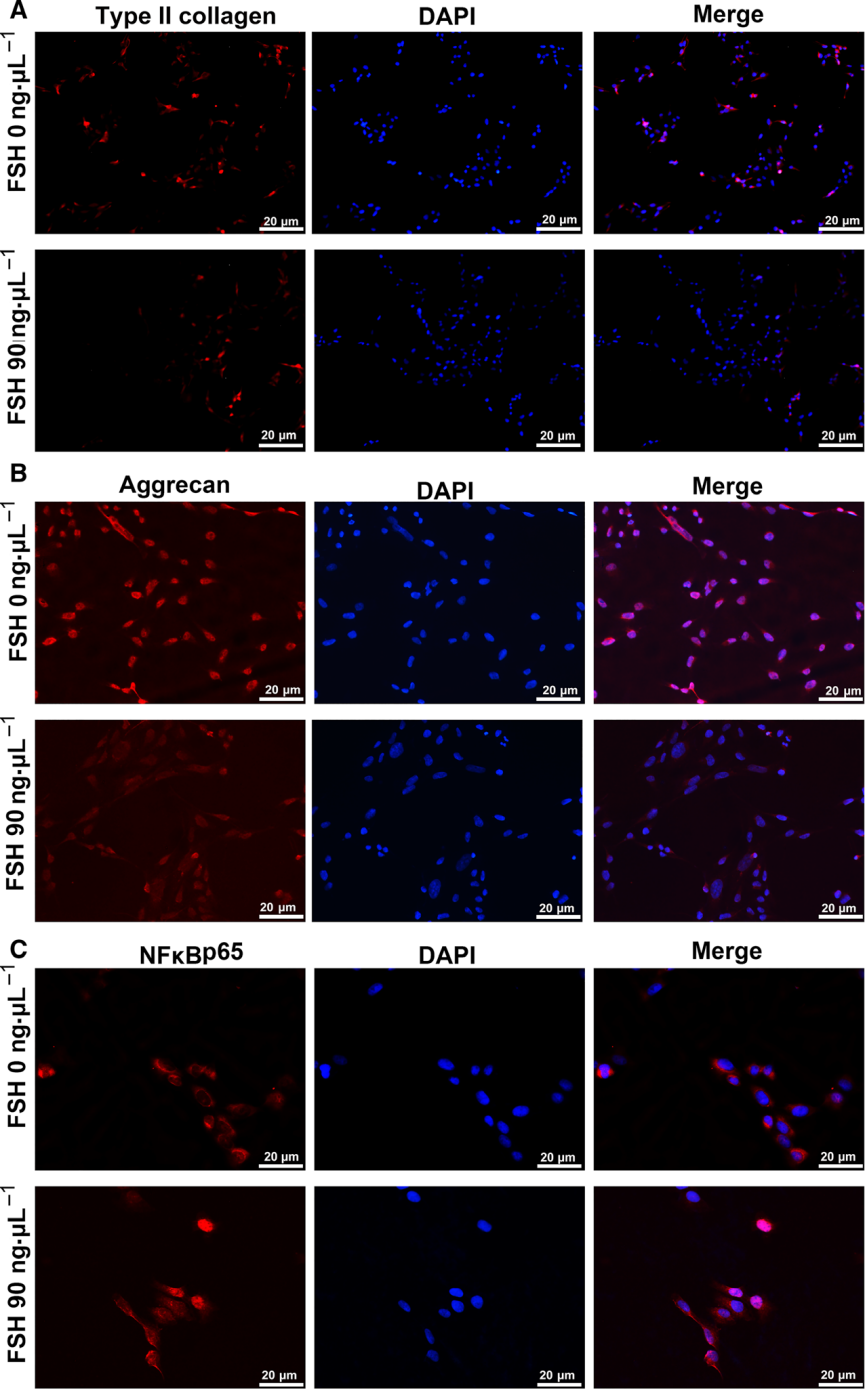
Immunofluorescence results showing the expression of type II collagen, aggrecan and NF‐κB p65 in ATDC5 chondrocytes treated with 0 and 90 ng µL^−1^ FSH. (A) Type II collagen; (B) aggrecan and (C) NF‐κB p65. Red represents type II collagen, aggrecan and NF‐κB p65 proteins (fluorescein isothiocyanate‐conjugated). Blue indicates the nucleus using 4',6‐dimidyl‐2‐phenylindole staining (×200) (*n* = 3). Scale bar = 20 μm.

One of the potential mechanisms of KOA progression is the degradation of cartilage extracellular matrix (ECM). Chondrocytes are responsible for the synthesis of ECM components, including type II collagen (collagen II) and aggrecan. Therefore, promoting collagen II and aggrecan expression in cartilage is a potential strategy for treating OA. In the present study, we examined the expression of type II collagen and aggrecan after ATDC5 chondrocytes were stimulated by different concentrations of FSH (0, 9, 30 and 90 ng·µL^−1^). As shown in Fig. 5A,B, 90 ng·µL^−1^ FSH significantly decreased the expression of type II collagen and aggrecan in ATDC5 chondrocytes.

**Fig. 6:**
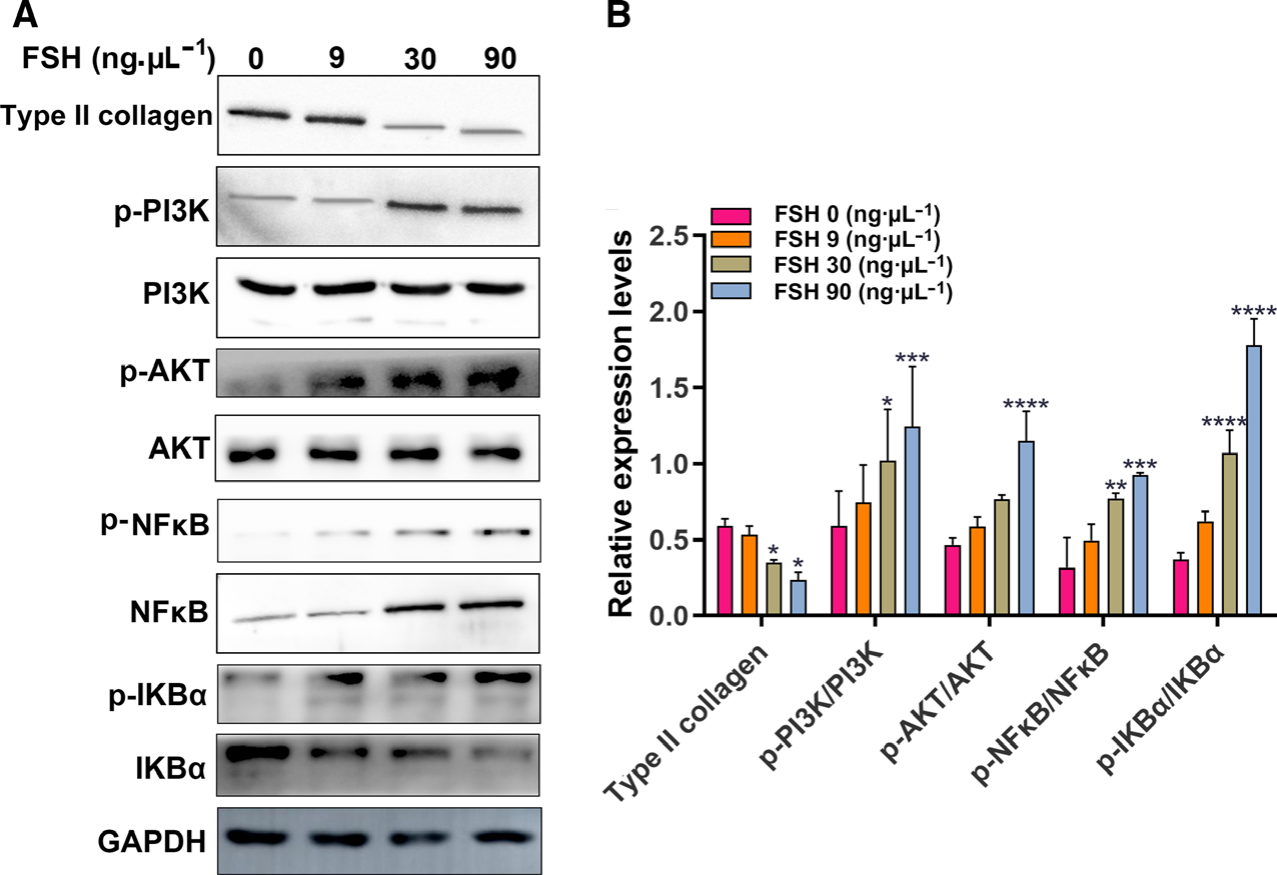
Effects of FSH at concentrations of 0, 9, 30 and 90 ng·µL^−1^ on type II collagen and the PI3K/AKT/NF‐κB signaling pathway in ATDC5 chondrocytes by western blotting. (A) Representative images of western blotting. (B) The protein expression levels of p‐PI3K/PI3K, p‐AKT/AKT, p‐NF‐κB/NF‐κB and p‐IKBα/IKBα. Each expression level was normalized with GAPDH (*n* = 3). Data are expressed as the SEM. Multiple comparisons were presented using one‐way ANOVA. *P < 0.05; **P < 0.01; ***P < 0.001 and ****P < 0.0001.

The nuclear translocation of NF‐κB p65 was also measured using confocal microscopic analysis. The results showed that 90 ng·μL^−1^ FSH stimulation in ATDC5 chondrocytes significantly promoted a dramatic increase in the translocation of p65 into the nucleus (Fig. 5C). Furthermore, the expression levels of type II collagen, p‐PI3K/PI3K, p‐AKT/AKT, p‐NF‐κB/NF‐κB and p‐IKBα/IKBα were detected in ATDC5 chondrocytes stimulated with FSH using western blotting. We found that FSH significantly decreased the expression of type II collagen in ATDC5 chondrocytes, in a concentration‐dependent manner (Fig. 6A,B). Rather, FSH significantly increased the expression levels of p‐PI3K/PI3K, p‐AKT/AKT, p‐NF‐κB/NF‐κB and p‐IKBα/IKBα in ATDC5 chondrocytes, with a concentration‐dependent manner (Fig. 6A,B). The above results suggested that high FSH level significantly decreased type II collagen and aggrecan expression and activated PI3K/AKT/NF‐κB pathway in chondrocytes.

## Discussion

In the present study, a high FSH level was found in menopausal female KOA patients with aged 50–60 years. Furthermore, FSH could promote the development of KOA through the PI3K/AKT/NF‐κB pathway. Thus, inhibition of FSH could become a novel treatment strategy for KOA.

An increase in FSH has been considered as a sign of menopause [9]. The serum FSH levels in Chinese adult women are low before the age of 40 years, then increase rapidly with age, and reach the highest levels at the age of 50–59 years [18]. However, FSH decreases with age from the age of 60 years. In the present study, postmenopausal KOA women were divided into three groups: 50–60 years age group, 61–70 years age group and > 70 years age group. Consistent with the results of previous studies, we found that levels of FSH decreased significantly with age. KOA women aged 50–60 years had the highest levels of FSH. The biochemical changes of articular cartilage usually occur before morphological degradation. Therefore, evaluation of the biochemical components of cartilage is of great value for the early diagnosis of KOA. The early damage of articular cartilage is mainly manifested by the loss of collagen and changes in the arrangement of collagen fibers. A T2 quantitative measurement is used to check the composition of knee cartilage in newly cartilage lesions and the surrounding cartilage 1–4 years before onset. The changes of cartilage components reflect the mechanism of cartilage degradation, and the diffuse and focal changes of cartilage components in the cartilage plate precede the development of cartilage lesions. In the present study, WOMAC scores and mean T2 values of postmenopausal female KOA patients significantly increased with age. We found that FSH might be a factor affecting the progression of OA. We further observed the effects of FSH on the symptoms and severity of postmenopausal KOA patients in the 50–60 years age group. All patients aged 50–60 years were divided into high FSH and low FSH groups. There were no statistical differences in BMI, E2, LDL‐C and WOMAC between the two groups. T2 value and TC levels were significantly higher in the high FSH group compared to the low FSH group. In comparison with the low FSH group, the pathological section of the high FSH group showed more severe cartilage damage. These results indicated that high FSH level could aggravate OA symptoms and injuries in the 50–60 years age group.

Studies have shown that activated PI3K/AKT pathway is closely related to the occurrence of chondrocyte apoptosis in OA. FSH is associated with the occurrence of various diseases by activating the PI3K/AKT signaling pathway [19, 20, 21]. For example, elevated FSH activates the PI3K/AKT signaling pathway and promotes the proliferation of ovarian cancer cells [22]. Several AKT substrates have been reported, including NF‐κB [23]. NF‐κB is a family of transcription factor proteins in which the p65 subunit has been found to be involved in inflammation, apoptosis and matrix degradation processes of chondrocytes [24, 25, 26, 27]. Currently, the NF‐κB pathway has been considered as a promising therapeutic target for the treatment of OA. NF‐κB is involved in many OA‐related events, including chondrocyte catabolism, chondrocyte survival and synovial inflammation [28, 29, 30]. In chondrocytes, NF‐κBp65 is retained in the cytoplasm by interacting with IκB inhibitory proteins. After stimulation, IκB is phosphorylated by IκB kinase and degraded by the proteasome, allowing free NF‐κBp65 to be transported to the nucleus, binding to NF‐κB response elements, thus inducing the transcription of related genes. In the present study, the expression levels of p‐PI3K/PI3K, p‐AKT/AKT and p‐NF‐κB/NF‐κB were distinctly increased in menopausal female KOA patients with high FSH and age 50–60 years. *In vitro*, with the increase of FSH concentration, the expression levels of p‐PI3K/PI3K, p‐AKT/AKT, p‐NF‐κB/NF‐κB and p‐IKBα/IKBα gradually increased, whereas the expression of type II collagen gradually decreased. Moreover, immunofluorescence staining results showed that, under FSH stimulation, NF‐κBp65 was significantly increased, whereas the expression levels of type II collagen and aggrecan were significantly decreased. Type II collagen and aggrecan are the main components of ECM, and KOA cartilages often occur the degradation of type II collagen and aggrecan [31, 32]. The hydrated structure of the matrix provides tensile and elastic strength to the articular cartilage, enabling the joint to maintain proper biomechanical functions. FSH stimulation could lead to cartilage erosion and degradation of ECM components in chondrocytes. In animal models, inhibition of NF‐κBp65 in the knee joint can reduce traumatic cartilage damage [33]. In chondrocytes, treatment with NF‐κB inhibitors reduces the expression of IL‐1β‐induced catabolic genes [34]. Thus, FSH could promote cartilage damage through the PI3K/AKT/NF‐κB pathway for menopausal female KOA patients with high FSH and age 50–60 years.

Our study suggests that the increase in FSH in postmenopausal women could be associated with the development of KOA. In addition, FSH might damage cartilage tissues through the PI3K/AKT/NF‐κB pathway. However, several limitations should be highlighted. First, a larger cohort is required to validate the correlation between FSH levels and postmenopausal women with KOA. Second, more research is needed with respect to exploring the PI3K/AKT/NF‐κB pathway in KOA. In future studies, we will continue to investigate new treatments for postmenopausal women with KOA by developing FSH inhibitors.

## Conclusions

In the present study, we found that FSH level was significantly increased in postmenopausal female KOA patients aged 50–60 years. Moreover, FSH might damage the cartilage tissues through the PI3K/AKT/NF‐κB pathway. These results indicate that FSH could be considered as a novel treatment strategy for postmenopausal female KOA patients aged 50–60 years.

## Conflict of interests

The authors declare that they have no conflicts of interest.

## Author contributions

JX conceived and designed the study. YL, MZ and DK conducted most of the experiments and data analysis, and wrote the manuscript. YW, JL, WL and YF participated in collecting data and helped to draft the manuscript. All authors reviewed and approved the final manuscript submitted for publication.

## Data Availability

The datasets analyzed during the current study are available from the corresponding author on reasonable request.
